# Metal Nanoparticles–Polymers Hybrid Materials I

**DOI:** 10.3390/polym14153117

**Published:** 2022-07-30

**Authors:** Iole Venditti

**Affiliations:** Sciences Department, Roma Tre University, Via della Vasca Navale 79, 00146 Rome, Italy; iole.venditti@uniroma3.it; Tel.: +39-06-5733-3388

Important discoveries have characterized the last decade, highlighting the importance of investment in research in fields such as medicine, biology, computer science, and physics [1,2,3,4,5,6,7,8,9].

Hybrid nanosystems, in which different components synergistically contribute to peculiar properties, are an important example of how multidisciplinarity is now essential for developing innovative ideas and materials. In particular, the study and development of hybrid materials based on metal nanoparticles and polymers require studies on the structure–property relationships, and will allow advanced applications in sectors such as energy, optoelectronics, and environmental protection [10,11,12,13,14,15,16].

Therefore, this Special Issue was born, collecting contributions from different sectors such as chemistry, engineering, physics, and biology. The great goal achieved with this collection is certainly the ability to provide an updated overview of both the great opportunities and the challenges that hybrid materials based on metal–polymer nanoparticles create.

One of the key topics of the last decade concerns the use of silver nanoparticles (AgNPs) and their increasing integration into environmental applications, including the monitoring and treatment of water pollution, which is raising concerns about their environmental impact. The review by Fiorati et al. [17] analyzes the situation based on the most recent literature and proposes a design strategy that combines better performance with the absence of risks for ecosystems. The review, starting with an overview of the recent preparations of AgNPs and their uses, indicates potential solutions based on AgNPs–cellulose hybrid materials that would be efficient and eco-compatible for monitoring and treating water pollution. The development of these hybrid materials following an eco-design approach can represent a winning strategy for exploiting AgNP-based nanotechnologies for the monitoring and treatment of water pollution.

I. Jacukowicz-Sobala et al. [18] presented the preparation of composite materials containing zero-valent copper (ZVC) dispersed in a matrix of two commercially available, strongly basic anion exchangers with a macroreticular (Amberlite IRA 900Cl) and gel-like (Amberlite IRA 402OH) structure. The Cu^0^ particles appeared in the resinous phase as a product of the reduction of the precursor, i.e., copper (I) oxide particles previously deposited in the two support materials. As a result, macroporous and gel-type hybrid products containing ZVC were obtained, with total copper contents of 7.7 and 5.3% by weight, respectively. X-ray diffraction and Fourier-transform infrared spectroscopy (FTIR) confirmed the successful transformation of the starting oxide particles into a metallic deposit. A scanning electron microscopy (SEM) study showed that the morphology of the deposit is mainly influenced by the type of matrix exchanger. Considering that both the reaction parameters and the operating techniques influence the size, shape, and distribution of Cu^0^, as well as the porous structure of the polymer matrix, the composite materials obtained can have promising applications, including in catalytic or photocatalytic chemical reactions involving both gaseous or liquid reagents, as well as showing antibacterial activity.

G. M. Di Salvo et al. [19] studied the self-assembly of amphiphilic diblock copolymers into polymeric vesicles, commonly known as polymersomes, resulting in a versatile system for a variety of applications including drug delivery and microreactors. In this study, the incorporation of hydrophobic plasmonic nanoparticles within a polymersome membrane facilitated the light-stimulated release of vesicle encapsulants. This work sought to achieve tunable, triggered release with non-invasive, spatiotemporal control using single-pulse irradiation. Gold nanoparticles (AuNPs) were incorporated as photosensitizers into the hydrophobic membranes of micron-scale polymersomes, and the cargo-release profile was controlled by varying the pulse energy and nanoparticle concentration. The study demonstrated the ability to achieve immediate vesicle rupture as well as vesicle poration resulting in cargo diffusion. Additionally, changing the pulse duration, from femtosecond to nanosecond, provided mechanistic insight into the photothermal and photomechanical contributors that govern membrane disruption in this polymer–nanoparticle hybrid system.

W. G. Lee et al. [20] presented the preparation of porous cellulose acetate (CA) for application as a battery separator. Cd(NO_3_)_2_·4H_2_O was utilized, with water pressure as an external physical force. When the CA was complexed with Cd(NO_3_)_2_·4H_2_O and exposed to external water pressure, the water flux through the CA was observed, indicating the generation of pores in the polymer. Furthermore, as the hydraulic pressure increased, the water flux increased proportionally, indicating the possibility of the control of the porosity and pore size. Surprisingly, the flux value, above 250 LMH (L/m^2^h), observed at the ratio of 1:0.35 (molar ratio of CA:Cd(NO_3_)_2_·4H_2_O) was higher than the fluxes of CA/other metal nitrate salt (Ni(NO_3_)_2_ and Mg(NO_3_)_2_) complexes. The higher value indicated that larger and abundant pores were generated in the cellulose acetate at the same water pressure. Thus, it could be postulated that the Cd(NO_3_)_2_·4H_2_O salt played a role as a stronger plasticizer than the other metal nitrate salts such as Ni(NO_3_)_2_ and Mg(NO_3_)_2_. The coordinative interactions between the CA and Cd(NO_3_)_2_·4H_2_O were investigated by IR spectroscopy. The change in ionic species in Cd(NO_3_)_2_·4H_2_O was analyzed by Raman spectroscopy.

Tissue engineering is an area in which biomedical research is investing heavily [21,22]. Tissue engineering represents an alternative to autologous grafts. Its application requires three-dimensional scaffolds, which mimic the human body’s environment. This topic is discussed by the work of J. Radwan-Pragłowska et al. [23] regarding bone-tissue engineering. In fact, bone is the second tissue to be replaced, and annually, over four million surgical treatments are performed. Bone tissue has a highly organized structure and contains mostly inorganic components. The scaffolds of the latest generation should not only be biocompatible but also promote osteoconduction. Poly (lactic acid) (PLA) nanofibers are commonly used for this purpose; however, they lack bioactivity and do not provide good cell adhesion. Chitosan is a commonly used biopolymer that positively affects osteoblasts’ behavior. The aim of this study was to prepare novel hybrid 3D scaffolds containing nanohydroxyapatite capable of stimulating cell responses. Matrices were successfully obtained by PLA electrospinning and microwave-assisted chitosan crosslinking, followed by doping with three types of metallic nanoparticles (Au, Pt, and TiO_2_). The products and semi-components were characterized in terms of their physicochemical properties, such as chemical structure, crystallinity, and swelling degree. The nanoparticles’ and ready biomaterials’ morphologies were investigated by SEM and TEM methods. Finally, the scaffolds were studied for their bioactivity on MG-63 and effects on current-stimulated biomineralization. The obtained results confirmed the preparation of tunable biomimicking matrices that may be used as a promising tool for bone-tissue engineering.

M. Wu et al. [24] introduced a three-dimensional (3D) pyramid to the polymer–plasmonic hybrid structure of polymethyl methacrylate (PMMA) composite AgNPs as a higher-quality, flexible surface-enhanced Raman scattering (SERS) substrate. The effective oscillation of light inside the pyramid valley could provide wide distributions of 3D “hotspots” in a large space. The inclined surface design of the pyramid structure could facilitate the aggregation of probe molecules, enabling the highly sensitive detection of rhodamine 6G (R6G) and crystal violet (CV). In addition, the AgNPs and PMMA composite structures provided a uniform space distribution for analyte detection in a designated hotspot zone. The incident light could penetrate the external PMMA film to trigger the localized plasmon resonance of the encapsulated AgNPs, achieving an enormous enhancement factor (~6.24 × 10^8^). After it underwent mechanical deformation, the flexible SERS substrate still maintained high mechanical stability, which was proved by experiments and theory. For practical applications, the prepared flexible SERS substrate was adapted for the in situ Raman detection of adenosine aqueous solution and methylene-blue (MB) molecule detection on the skin of a fish, providing a direct and nondestructive active platform for detection on surfaces with any arbitrary morphology and aqueous solution.

Magnetic nanoparticles (MNPs) are largely investigated for biomedical applications. However, if not properly designed, MNPs can cause several problems, such as cytotoxicity or hemolysis. J.A. Roacho-Pérez et al. [25] compared bare magnetite nanoparticles against magnetite nanoparticles coated with a combination of polyethylene glycol 3350 (PEG 3350) and polysorbate 80 (Tween 80). The physical characteristics of the nanoparticles were evaluated. A primary culture of sheep adipose mesenchymal stem cells was developed to measure the nanoparticle cytotoxicity. A sample of erythrocytes from a healthy donor was used for the hemolysis assay. The results showed the successful obtention of magnetite nanoparticles coated with PEG 3350–Tween 80, with a spherical shape. Biological studies showed a lack of cytotoxicity at doses lower than 1000 µg/mL for mesenchymal stem cells, and a lack of hemolytic activity at doses lower than 100 µg/mL for erythrocytes.

At the end of this Special Issue, we have the work of P. Shao et al. [26] addressing the effects of cuprous nano-oxide on the flame-retardant properties of calcium alginate. Nanocuprous oxide was prepared on a sodium alginate template from which Cl^−^ was excluded and based on which calcium alginate/nanocuprous oxide hybrid materials were prepared by a Ca^2+^-crosslinking and freeze-drying process. The thermal degradation and combustion behavior of the materials were studied by related characterization techniques using pure calcium alginate for comparison. The results show that the weight loss rate, heat release rate, peak heat release rate, total heat release rate, and specific extinction area of the hybrid materials were remarkably lower than those of pure calcium alginate, and the flame-retardant performance was significantly improved. In fact, nanocuprous oxide formed a dense protective layer of copper oxide, calcium carbonate, and carbon by lowering the initial degradation temperature for the polysaccharide chain during thermal degradation and catalytically dehydrating to char in the combustion process; thereby, it could isolate combustible gases, increase carbon residual rates, and notably reduce heat release and smoke evacuation.

In conclusion, as the editor of this Special Issue, I am aware that the diversity and innovation of new compounds and tools that are rapidly developing in the field of multidisciplinary research related to nanomaterials based on noble metals cannot all be collected in a single volume. However, I am certain that this collection will contribute to the interest in the research in this area, providing our readers with a broad and updated overview of this topic.
